# Effect of neoadjuvant chemotherapy on survival in patients with T1 high-grade non-muscle-invasive bladder cancer who underwent radical cystectomy

**DOI:** 10.1097/MD.0000000000034501

**Published:** 2023-08-04

**Authors:** Long Huang, Kang Jia, Kai Yao, Dongliang Liu, Yan Xu, Quanda Liu

**Affiliations:** a Department of Urology, Chengdu, China.

**Keywords:** neoadjuvant chemotherapy, non-muscle-invasive bladder cancer, overall survival, progression risk, radical cystectomy

## Abstract

Patients with non-muscle-invasive bladder cancer (NMIBC) who are at high and very high risk of disease progression are recommended for radical cystectomy (RC). However, the impact of neoadjuvant chemotherapy (NAC) on survival outcomes in NMIBC patients undergoing RC remains unclear. Patients diagnosed with T1 high-grade NMIBC who underwent RC were identified from the Surveillance, Epidemiology, and End Results (SEER) database. Overall survival (OS) was assessed using the Kaplan–Meier technique, and multivariable Cox regression analysis was conducted to determine the independent factors of OS. A total of 1268 T1 high-grade NMIBC patients who underwent RC between 2004 and 2015 were included in the study. NAC was administered to 76 (6.0%) patients. At a median follow-up of 75 months, there was no significant difference in the OS between the NAC and non-NAC groups (HR = 0.89, 95% CI 0.61–1.30, *P* = .539). However, in the multivariate Cox regression model, NAC demonstrated a more pronounced improvement in OS approaching statistical significance (HR = 0.7, 95% CI 0.47–1.05, *P* = .088). Subgroup analysis revealed a survival benefit of NAC in patients with lymph node metastasis. In summary, the results of this study suggest that NAC has the potential to confer a survival advantage in patients diagnosed with T1 high-grade NMIBC who undergo RC, but additional studies are needed. Nonetheless, the survival benefits of NAC in patients with lymph node involvement are apparent.

## 1. Introduction

Bladder cancer is the 10th most commonly diagnosed malignancy worldwide, and its incidence rises to 6th when considering men only.^[1]^ Non-muscle-invasive bladder cancer (NMIBC), characterized by tumor confinement to the mucosa and submucosa, accounts for approximately 75% of all bladder cancer cases.^[2]^ According to the guidelines of the European Association of Urology (EAU), the choice of treatment strategy for NMIBC is based on the patient risk of disease progression.^[3]^ Typically, for low-risk and intermediate-risk patients, transurethral resection of the bladder tumor (TURBT) along with intravesical instillation therapy is recommended. However, for high-risk and very high-risk patients, TURBT combined with Bacillus Calmette-Guérin instillation therapy is recommended, with radical cystectomy (RC) also considered as an option, particularly for those with a very high risk of disease progression.

Numerous studies have consistently demonstrated the survival benefits of neoadjuvant chemotherapy (NAC) in patients with muscle-invasive bladder cancer (MIBC) who undergo RC.^[4–6]^ As a result, NAC has been included as a recommended treatment option for MIBC in major clinical guidelines.^[7–9]^ These guidelines, which provide evidence-based recommendations for optimal patient management, emphasize the importance of NAC in improving outcomes for MIBC patients. Approximately 50% of cases of T1G3 NMIBC were found to be upstaged to MIBC after RC, with 16.3% of cases presenting lymph node metastasis.^[10]^ Notably, lymph node metastasis has been strongly associated with the occurrence of distant metastasis.^[11]^ Given the substantial likelihood of T1G3 NMIBC progressing to MIBC and the presence of lymph node metastases, it is plausible that NAC may offer survival advantages in this specific patient population. However, to date, no studies have investigated the impact of NAC on the survival outcomes of patients with NMIBC who underwent RC. Further research is warranted to evaluate the potential benefits of NAC in this context.

Therefore, in this study, based on the National Cancer Institute Surveillance, Epidemiology, and End Results (SEER) database, we aimed to explore the impact of NAC on overall survival (OS) in patients with T1 high-grade NMIBC who underwent RC, as these patients belonged to the high-risk or very high-risk groups.

## 2. Patients and methods

### 2.1. Patients and covariates

**Figure 1.**
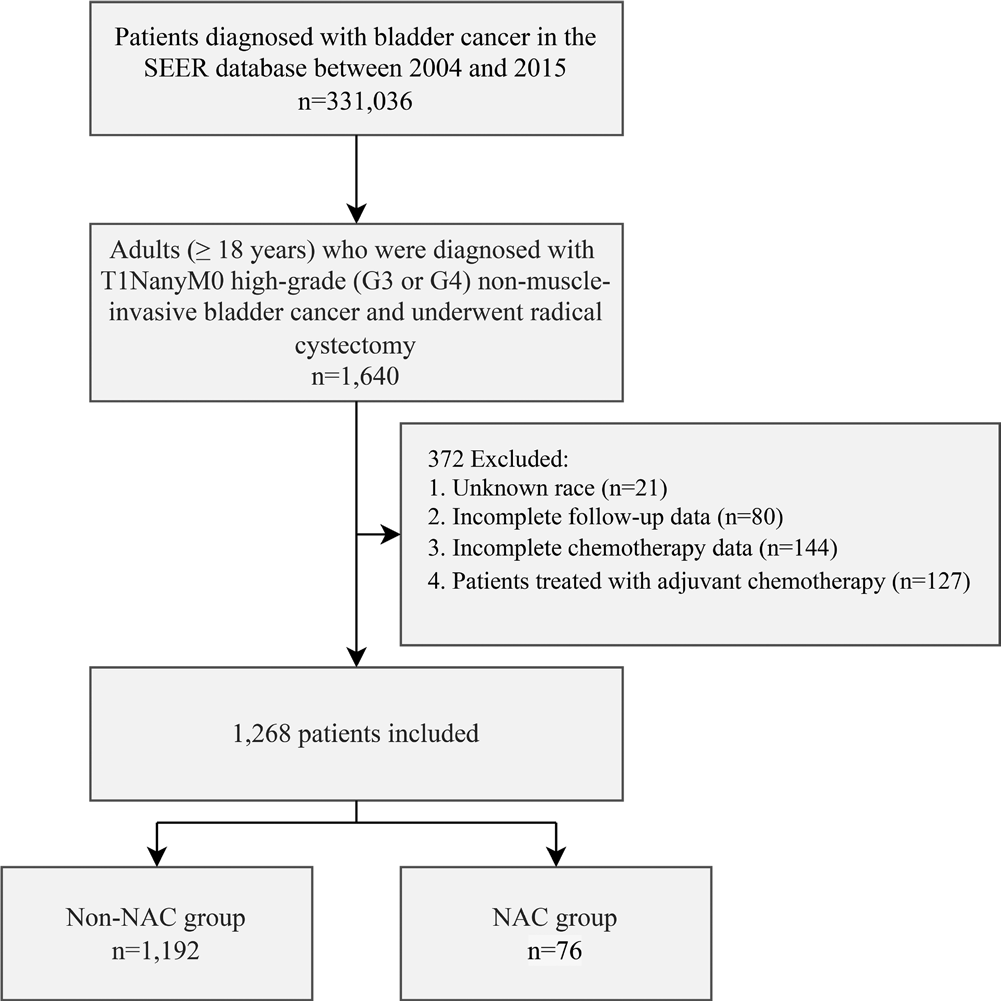
CONSORT diagram.

Patients who were diagnosed with NMIBC and subsequently underwent RC between 2004 and 2015 were identified in the SEER database. Inclusion criteria for our study consisted of individuals aged 18 years or older with high-grade tumors (G3 or G4) and T1NanyM0 stage according to the 6th edition of the American Joint Committee on Cancer TNM staging system. Exclusion criteria encompassed patients with incomplete follow-up data (including insufficient information on follow-up duration and vital status), unknown race, and inadequate details regarding systemic chemotherapy (specifically, the implementation and treatment sequence with RC). Additionally, patients who received adjuvant chemotherapy were excluded from the study. This study included both urothelial and non-urothelial carcinomas. The screening process is illustrated in Figure 1.

Clinical variables assessed in this study encompassed age, sex, race, N stage, tumor size, and histology. According to the risk stratification model proposed by the EAU in 2021, age over 70 years, presence of multiple tumors, and tumor diameter of 3 cm or larger are recognized as risk factors for disease progression.^[12]^ Consequently, in this study, age was categorized into 2 groups: ≤70 and >70 years, while tumor size was classified as <3 cm, ≥3 cm, or unknown. Race was divided into white and other categories, N stage into N0 and N+, and tumor histology into urothelial carcinoma (codes 8050, 8120, and 8130) and non-urothelial carcinoma based on the SEER database codes. The administration of systemic chemotherapy for each patient, along with its timing in relation to surgery (before surgery, after surgery, or both), was recorded in the SEER database. In cases where patients received systemic chemotherapy prior to surgery, it was considered NAC. Based on the receipt of NAC, patients were then divided into 2 groups: the NAC group and the non-NAC group.

### 2.2. Statistical analysis

Continuous and categorical variables were compared between the NAC and non-NAC groups using the Mann–Whitney *U* test and chi-square test, respectively. The Kaplan–Meier method was employed to estimate OS for both the NAC and non-NAC groups, and the log-rank test was utilized to assess the statistical significance of the observed differences between the 2 groups. Subgroup analyses were conducted based on predetermined demographic and oncological characteristics. Finally, univariate and multivariate Cox regression analyses were used to identify independent predictors of OS, with all variables included in the multivariate analysis, regardless of their significance in the univariate analysis.

## 3. Results

### 3.1. Patient characteristics

A total of 1268 patients diagnosed with T1 high-grade NMIBC who underwent RC with or without NAC between 2004 and 2015 were included in this study. Among them, 1192 patients (94.0%) did not receive NAC, while 76 patients (6.0%) received NAC. The median follow-up duration was 75 months (interquartile range, 42–116 months). The median age of the patients was 68 years (interquartile range, 61–75 years). The majority of the patients were aged 70 years or younger (59.1%), male (81.2%), white (89.4%), presented with urothelial carcinoma (95.1%), and had no lymph node metastasis (97.6%). The distribution of tumor size was 26.4% for tumors smaller than 3 cm, 27.4% for tumors 3 cm or larger, and 46.1% for cases with unknown tumor size. When comparing the NAC group to the non-NAC group, a higher proportion of patients in the NAC group had lymph node metastasis (9.2% vs 2.0%, *P* < .001), and a lower proportion had urothelial carcinoma (88.2% vs 95.6%, *P* = .009). However, there were no significant differences between the 2 groups in terms of age, sex, race, or tumor size (all *P* > .05). The clinical characteristics of the patients are summarized in Table 1.

**Table 1:** Patient baseline characteristics.

Variables	Total, No. (%)	Non-NAC, No. (%)	NAC, No. (%)	*P* value
Age, yr (median, IQR)	68 (61, 75)	69 (61, 75)	67 (59, 72)	.073
Age, yr				.224
≤70	750 (59.1%)	700 (58.7%)	50 (65.8%)	
>70	518 (40.9%)	492 (41.3%)	26 (34.2%)	
Sex				.418
Male	1029 (81.2%)	970 (81.4%)	59 (77.6%)	
Female	239 (18.8%)	222 (18.6%)	17 (22.4%)	
Race				.121
White	1134 (89.4%)	1062 (89.1%)	72 (94.7%)	
Other	134 (10.6%)	130 (10.9%)	4 (5.3%)	
N stage				<.001
N0	1237 (97.6%)	1168 (98.0%)	69 (90.8%)	
N+	31 (2.4%)	24 (2.0%)	7 (9.2%)	
Size, mm				.779
<30	335 (26.4%)	316 (26.5%)	19 (25%)	
≥30	348 (27.4%)	329 (27.6%)	19 (25%)	
Unknown	585 (46.1%)	547 (45.9%)	38 (50%)	
Histology				.009
Urothelial	1206 (95.1%)	1139 (95.6%)	67 (88.2%)	
Non-urothelial	62 (4.9%)	53 (4.4%)	9 (11.8%)	

IQR = interquartile range, NAC = neoadjuvant chemotherapy.

### 3.2. Overall survival

**Figure 2.**
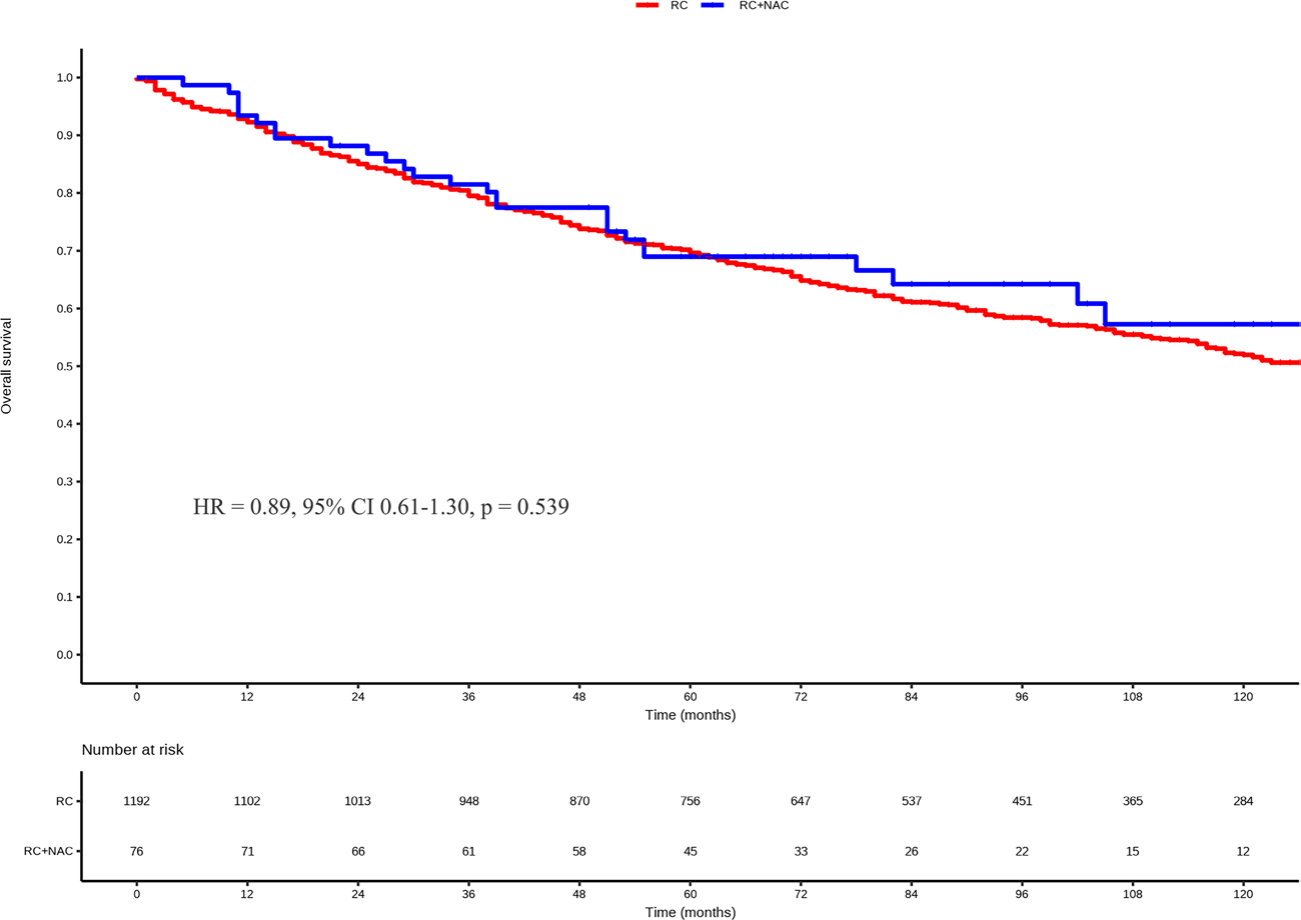
Kaplan–Meier survival curves in the NAC group and the non-NAC group. NAC = neoadjuvant chemotherapy.

**Figure 3.**
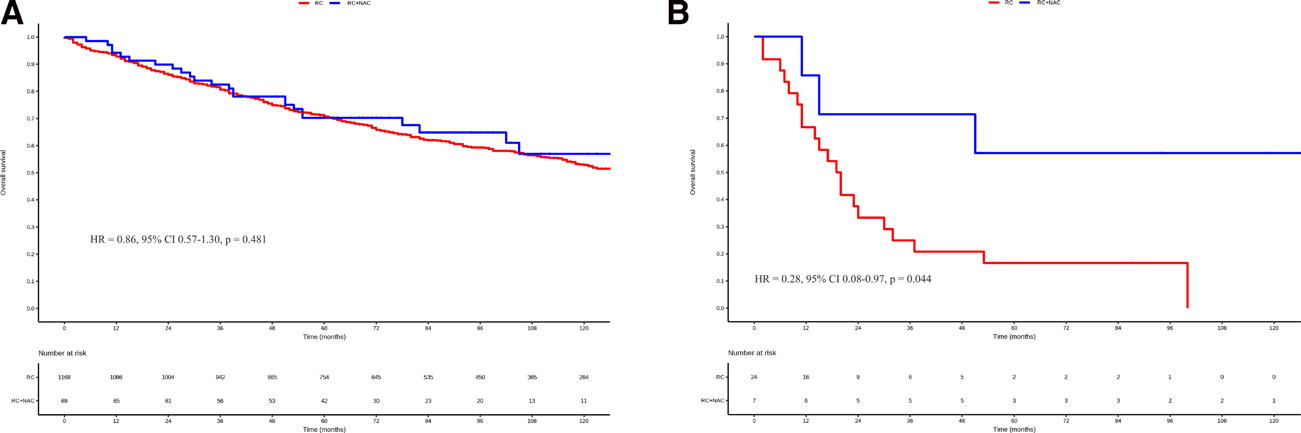
Kaplan–Meier survival curves depicting the impact of neoadjuvant chemotherapy (NAC) on overall survival in the N0 and N + groups.

**Figure 4.**
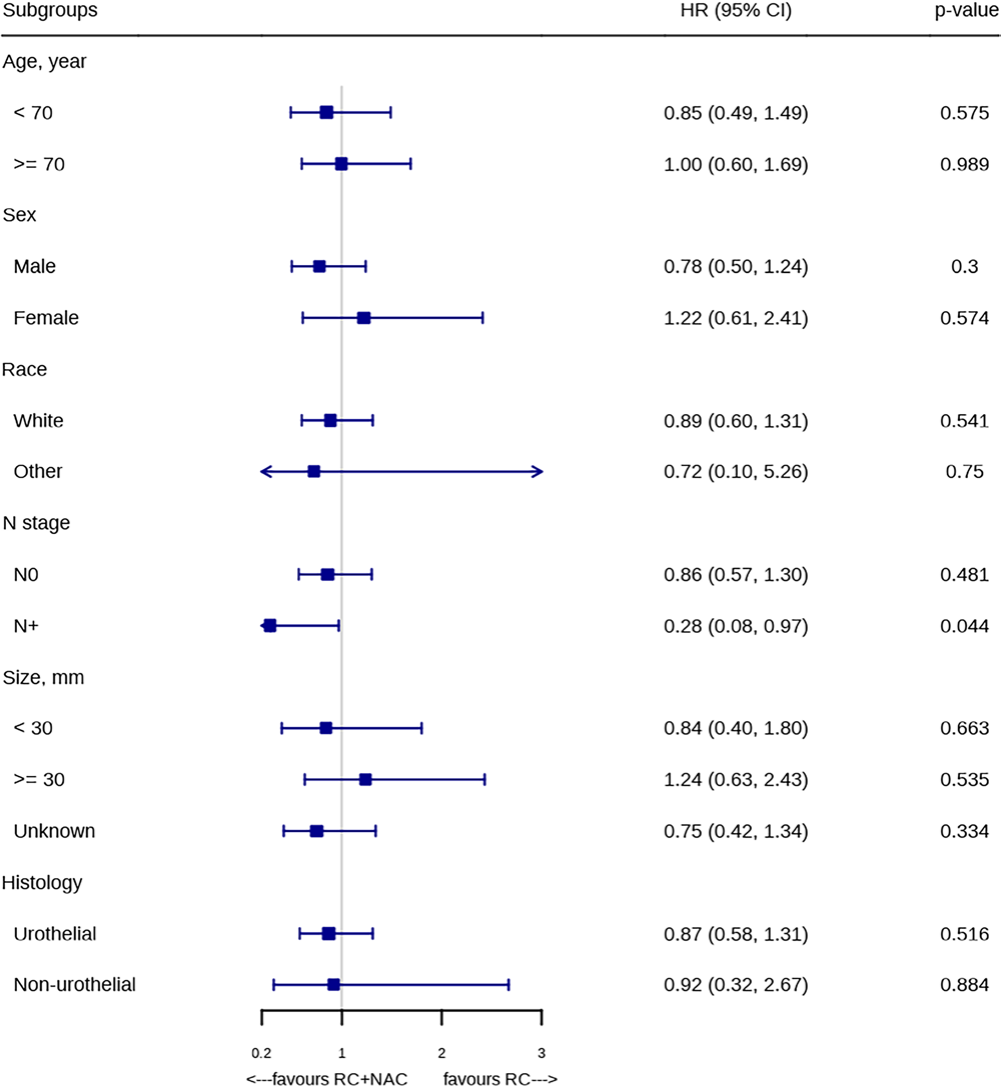
Treatment effect of neoadjuvant chemotherapy (NAC) in all prespecified subgroups.

At a median follow-up of 75 months, there was no significant difference in the OS between the NAC and non-NAC groups (HR = 0.89, 95% CI 0.61–1.30, *P* = .539, Fig. 2). In subgroup analyses, when patients were stratified into N0 and N + categories, NAC did not result in improved OS compared to non-NAC in N0 patients (HR = 0.86, 95% CI 0.57–1.30, *P* = .481, Fig. 3A). However, in N1 patients, NAC led to a significant improvement in OS (HR = 0.28, 95% CI 0.08–0.97, *P* = .044, Fig. 3B). In subgroup analyses based on other pre-defined variables, NAC did not significantly improve OS compared to non-NAC (all *P* > .05, Fig. 4).

Finally, univariate and multivariate Cox regression analyses were performed (Table 2). Older age (HR = 2.30, 95% CI 1.95–2.71, *P* < .001) and lymph node metastasis (HR = 3.95, 95% CI 2.57–6.06, *P* < .001) were identified as independent risk factors. Additionally, NAC demonstrated a more pronounced improvement in OS (HR = 0.7, 95% CI 0.47–1.05, *P* = .088), approaching statistical significance.

**Table 2:** Univariate and multivariate Cox model analyses.

Variables	Univariate Cox regression		Multivariate Cox regression	
	HR (95% CI)	*P* value	HR (95% CI)	*P* value
Age, yr				
≤70	reference		reference	
>70	2.33 (1.98, 2.75)	<.001	2.30 (1.95, 2.71)	<.001
Sex				
Male	reference		reference	
Female	1.06 (0.86, 1.30)	.584	1.05 (0.85, 1.30)	.632
Race				
White	reference		reference	
Other	0.86 (0.65, 1.14)	.298	0.88 (0.66, 1.16)	.366
N stage				
N0	reference		reference	
N+	3.82 (2.55, 5.72)	<.001	3.95 (2.57, 6.06)	<.001
Size, mm				
<30	reference		reference	
≥30	1.04 (0.84, 1.30)	.7	1.04 (0.83, 1.29)	.756
Unknown	0.97 (0.79, 1.18)	.759	0.97 (0.79, 1.18)	.759
Histology				
Urothelial	reference		reference	
Non-urothelial	1.24 (0.85, 1.81)	.272	1.13 (0.76, 1.66)	.544
NAC				
No	reference		reference	
Yes	0.89 (0.61, 1.30)	.539	0.70 (0.47, 1.05)	.088

NAC, neoadjuvant chemotherapy.

## 4. Discussion

According to the 2021 EAU risk stratification model, NMIBC patients are classified into 4 groups based on the risk of disease progression: low, intermediate, high, and very high. The 10-year probability of disease progression ranges from 3.7% (95% CI 2.3%–5.9%) for the low-risk group to 53% (95% CI 36%–73%) for the very high-risk group. For the high-risk and very high-risk groups, RC is recommended as one of the treatment options, particularly for patients in the very high-risk category.^[12]^ This recommendation is based on the potential for understaging after TURBT and the poorer prognosis associated with the progression of NMIBC to MIBC compared to an initial diagnosis of MIBC.^[10,13,14]^

Several randomized controlled trials have confirmed the survival benefits associated with platinum-based NAC in MIBC patients undergoing RC.^[4–6]^ The underlying mechanisms of these benefits encompass tumor downstaging, facilitated by NAC, which improves the feasibility of subsequent surgery, as well as the eradication of potential micrometastases.^[7]^ Lymph node metastasis is the most common type of metastasis and is strongly associated with distant metastasis in patients with MIBC. In an autopsy study, Wallmeroth et al found that 59% of patients with MIBC had lymph node metastasis, and there was a positive association between lymph node metastasis and distant metastasis (*P* < .0001).^[11]^ In NMIBC, the probability of lymph node metastasis is also not negligible.^[10,15–17]^ In a retrospective study involving 219 NMIBC patients, 15% of the patients had lymph node metastasis, and the probability of metastasis was positively correlated with the number of TURBT procedures and tumor upgrading after RC.^[17]^ In another multicenter study involving 1136 patients diagnosed with urothelial carcinoma, Fritsche et al found that 15.6% of cT1G3 patients had lymph node metastasis, and 35.5% of patients died from metastatic disease during a median follow-up of 48 months.^[10]^ The high probability of lymph node metastasis and poor prognosis suggest a potential role for NAC in (very) high-risk NMIBC patients who underwent RC. However, no studies have investigated the role of NAC in patients with NMIBC who have undergone RC.

Based on the 2021 EAU risk stratification model, all T1G3 NMIBC patients are classified into high-risk or very high-risk groups, for which RC is recommended, particularly for individuals categorized as very high-risk of progression.^[12]^ In this study, we investigated the impact of NAC on the survival of patients with T1 high-grade NMIBC who underwent RC, based on the SEER database. Our findings founded that NAC did not result in survival benefits in the overall population. However, in the multivariable Cox regression model, NAC demonstrated a more pronounced improvement in OS (HR = 0.7, 95% CI 0.47–1.05, *P* = .088), approaching statistical significance. Additionally, it is important to consider the relatively low lymph node metastasis rate (4.1%) observed in our study compared to previous research, which may have led to an underestimation of the effect of NAC.^[10,15–17]^ Another factor that needs to be taken into consideration is the relatively small sample size (76 patients received NAC) and its potential impact on statistical power. In conclusion, NAC may have potential benefits for T1 high-grade NMIBC patients undergoing RC, but further research is warranted. Nonetheless, the survival benefits of NAC in patients with lymph node involvement are evident.

In the present study, lymph node metastasis was identified as an independent prognostic risk factor for survival, emphasizing the essential role of lymph node dissection in NMIBC patients by indirectly improving outcomes by clarifying prognosis and guiding treatment as well as directly enhancing outcomes through its therapeutic effect, as shown in several previous studies.^[18,19]^

It is essential to acknowledge that our study possesses several strengths and limitations. Firstly, the utilization of the SEER database allowed us to examine a large, population-based cohort of patients, enhancing the generalizability of our findings. Another strength is that, given the limited use of NAC in NMIBC patients, there is currently no existing study exploring its impact specifically on high-risk NMIBC patients who underwent RC. To the best of our knowledge, this is the first study to investigate the role of NAC in patients with NMIBC who have undergone RC. Nonetheless, certain limitations of this study must be addressed. Firstly, the retrospective nature of the study may have introduced selection bias. Secondly, the SEER database lacks detailed information on the chemotherapy regimens, doses, and treatment compliance, making it challenging to assess the effect of specific chemotherapy protocols on survival outcomes. Additionally, the relatively small sample size of patients who received NAC in our study may have decreased the statistical power of the analysis. Future prospective studies with comprehensive chemotherapy data are warranted to elucidate the optimal chemotherapy strategy for (very) high-risk patients with NMIBC who undergo RC.

## 5. Conclusion

In conclusion, this population-based study suggests that NAC may offer potential survival benefits for patients with T1 high-grade NMIBC who undergo RC, but further research is warranted. Nevertheless, the survival advantages of NAC in patients with lymph node invasion are evident.

## Acknowledgments

We thank the Surveillance, Epidemiology, and End Results databases for providing the high-quality data.

## Author contributions

**Conceptualization:** Long Huang, Kang Jia, Kai Yao, Yan Xu, Quanda Liu.

**Data curation:** Long Huang, Kang Jia, Kai Yao, Quanda Liu.

**Formal analysis:** Long Huang, Quanda Liu.

**Methodology:** Dongliang Liu.

**Software:** Yan Xu.

**Supervision:** Quanda Liu.

**Validation:** Long Huang.

**Writing – original draft:** Long Huang, Kang Jia.

**Writing – review & editing:** Quanda Liu.
